# The antibacterial toxin colicin N binds to the inner core of lipopolysaccharide and close to its translocator protein

**DOI:** 10.1111/mmi.12568

**Published:** 2014-03-28

**Authors:** Christopher L. Johnson, Helen Ridley, Roberta Marchetti, Alba Silipo, David C. Griffin, Lucy Crawford, Boyan Bonev, Antonio Molinaro, Jeremy H. Lakey

**Affiliations:** ^1^ Centre for Bacterial Cell BiologyInstitute for Cell and Molecular BiosciencesFaculty of Medical SciencesNewcastle UniversityFramlington PlaceNewcastle‐upon‐TyneNE2 4HHUK; ^2^ Department of Chemical SciencesUniversity of Naples Federico IIVia Cinthia 480126NapoliItaly; ^3^ School of Life SciencesUniversity of Nottingham, Queens Medical CentreNottinghamNG7 2UHUK

## Abstract

Colicins are a diverse family of large antibacterial protein toxins, secreted by and active against *E**scherichia coli* and must cross their target cell's outer membrane barrier to kill. To achieve this, most colicins require an abundant porin (e.g. OmpF) plus a low‐copy‐number, high‐affinity, outer membrane protein receptor (e.g. BtuB). Recently, genetic screens have suggested that colicin N (ColN), which has no high‐affinity receptor, targets highly abundant lipopolysaccharide (LPS) instead. Here we reveal the details of this interaction and demonstrate that the ColN receptor‐binding domain (ColN‐R) binds to a specific region of LPS close to the membrane surface. Data from *in vitro* studies using calorimetry and both liquid‐ and solid‐state NMR reveal the interactions behind the *in vivo* requirement for a defined oligosaccharide region of LPS. Delipidated LPS (LPS^Δ^^LIPID^) shows weaker binding; and thus full affinity requires the lipid component. The site of LPS binding means that ColN will preferably bind at the interface and thus position itself close to the surface of its translocon component, OmpF. ColN is, currently, unique among colicins in requiring LPS and, combined with previous data, this implies that the ColN translocon is distinct from those of other known colicins.

## Introduction

**Figure 1:**
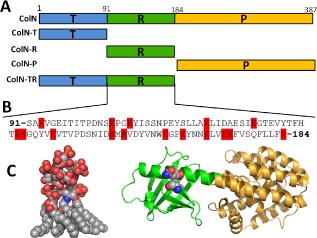
Domain structure of ColN. A. Schematic diagram of ColN highlighting T‐domain (blue), R‐domain (green) and P‐domain (amber) and their abbreviated construct names in ColN. B. Amino acid sequence of ColN‐R (91–184 aa) highlighting positively charged amino acids in red. C. PDB structures of (a) ColN (1A87) colouring as above and missing the disordered 90 residues of the T‐domain. Showing the residues mutated to cysteines to form the disulphide bridge (V94 and Q179) as space filling. Also at the same scale a representative LPS structure equivalent to Rd plus Hep(III) (see Fig. 4) from the structure of the TLR4‐MD‐2‐*E. coli* LPS complex 3FXI (Park *et al*., 2009).

Colicins are a family of highly effective bactericidal proteins which are produced by, and toxic to, related strains of *Escherichia coli* (*E. coli*). They consist of three functional domains, an N‐terminal translocation (T) domain, a central receptor binding (R) domain and a C‐terminal domain which carries the lethal activity (Fig. 1A). ColN belongs to the pore‐forming toxin family which insert their C‐terminal pore‐forming (P) domains into the inner membrane, forming voltage‐gated channels which result in cell death (Pugsley, 1987; Bourdineaud *et al*., 1990a; Fourel *et al*., 1990; El‐Kouhen *et al*., 1993; El Kouhen and Pages, 1996). Other lethal activities of colicins include DNase or RNase activity or inhibition of peptidoglycan biosynthesis (for more extensive reviews see Cascales *et al*., 2007; Jakes and Cramer, 2012). In order to exert this lethal activity, colicins hijack a range of outer membrane proteins (OMPs) whose normal function is the transport of nutrients across the outer membrane. An emerging theme in colicin biology is the exploitation of a pair of OMPs. These may be two different OMPs, e.g. BtuB and OmpF used by ColE2–9 (Cascales *et al*., 2007) or a pair of the same type of receptor, e.g. Cir used by Colicin Ia (ColIa) (Jakes and Finkelstein, 2010). Most E‐type colicins are proposed to utilize BtuB as their primary high‐affinity receptor. The interaction between BtuB and the R‐domains of ColE3 (Kurisu *et al*., 2003), ColE2 (Sharma *et al*., 2007) and ColE9 (Penfold *et al*., 2000) is tight, with measured *Kd*s of ∼ 1 nM. ColE2 and E3 have similar R‐domain sequences, possessing an extended ∼ 100 Å coiled‐coil R‐domain which, once bound to BtuB, places the colicin at an angle of ∼ 45° relative to the membrane plane (Kurisu *et al*., 2003; Sharma *et al*., 2007). This bound orientation is then proposed to facilitate the search for their secondary receptor OmpF which is used as a translocator (Housden and Kleanthous, 2012; Jakes and Cramer, 2012). In the case of ColE9, two OmpF binding sites (OBS1 & 2 ) have been identified in the intrinsically disordered T‐domain which are proposed to guide the colicin through the OmpF channel (Housden *et al*., 2010; 2013). In the case of ColIa, it has been elegantly demonstrated that it utilizes two identical copies of its OMP receptor Cir; one as a receptor the other as a translocator (Jakes and Finkelstein, 2010; Jakes and Cramer, 2012). ColIa is proposed to bind to its primary Cir receptor by a high‐affinity R‐domain mediated interaction. Once docked, ColIa is believed to seek out a second copy of Cir which it uses as a translocator, presumably by an as yet unidentified Cir binding site in T‐domain (Jakes and Finkelstein, 2010; Jakes and Cramer, 2012). ColN, like ColIa, utilizes and binds to a single type of OMP, OmpF, as its receptor (Evans *et al*., 1996a) but its R‐domain (ColN‐R) has a very weak affinity for OmpF (Evans *et al*., 1996b). This situation was recently clarified as it has been shown that ColN, like ColE9, contains an OBS in its flexible T‐domain (ColN‐T) (Johnson *et al*., 2013). The *Kd* measured for ColN‐T binding to OmpF was found to be ∼ 3 μM, similar to the 2 μM and 24 μM measured for OBS1 and OBS2 respectively of ColE9 (Housden *et al*., 2010).

**Figure 2:**
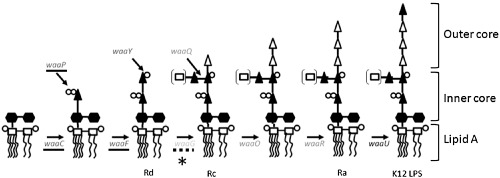
Simplified pathway for the biosynthesis of LPS in *E**. coli* LPS is synthesized from left to right by‐products of the genes shown in italics. Lipid A is composed of glucosamine (open squares) and acyl chains (black lines). Filled hexagons = KDO, filled triangles = heptose, open triangles = glucose, open circles = phosphate, double open circles = pyrophosphates. Genes found by Sharma *et al*. (2009) to be required for toxicity are underlined (waaC, waaP and waaF) while those not required are not (waaQ, waaO and waaR). waaU is not represented in the Keio collection. The gene found to render the cells partially sensitive to ColN (waaG) is underlined with dots while * = GalU is also involved in the addition of glucose however is not shown due its leaky phenotype. The nomenclature to which the LPS structures correspond is written underneath where appropriate.

Previously a genome‐wide screen (Sharma *et al*., 2009) of colicin activity against mutants from the Keio Collection (Baba *et al*., 2006) demonstrated that only ColN toxicity requires a minimum length of LPS on target cells (Fig. 2). The authors propose that the partial phenotype of the *waaG* and *galU* knockouts indicates that ColN interacts with the LPS inner core plus the first added glucose, as presented in Rc LPS (Sharma *et al*., 2009). LPS would serve as an ideal outer membrane receptor for ColN as there are estimated to be ∼ 1 million copies per cell covering ∼ 75% of the bacterial surface (Raetz and Whitfield, 2002). This compares favourably to ∼ 200 copies of BtuB per cell, ∼ 7000 copies of Cir and ∼ 100 000 copies of OmpF. Additionally, as OmpF is known to be in complex with LPS in the outer membrane (Baboolal *et al*., 2008), interaction with LPS‐bound to OmpF would serve to orientate ColN precisely beside its dedicated translocator. Presumably, as ColN is the smallest colicin and does not possess an extended coiled‐coil R‐domain like Col Ia/E3/E9 this will be reflected in its receptor usage.

Here we demonstrate that it is ColN‐R (Fig. 1B and C) which is responsible for binding LPS (Fig. 1C). Isothermal Titration Calorimetry (ITC), Saturation Transfer Difference NMR (STD‐NMR), solid‐state ^31^P magic angle spinning NMR (^31^PMAS‐NMR) and real‐time cell killing assays show that it is the terminal glucose and heptose moieties and phosphates which are recognized by ColN‐R. The data also suggest that, while the sugar moieties of LPS determine specificity, the lipid component is critical for affinity. ColN binds LPS in the membrane interfacial zone which may orient it correctly for the next stage in translocation.

## Results

### ColN‐R is responsible for LPS binding

**Figure 3:**
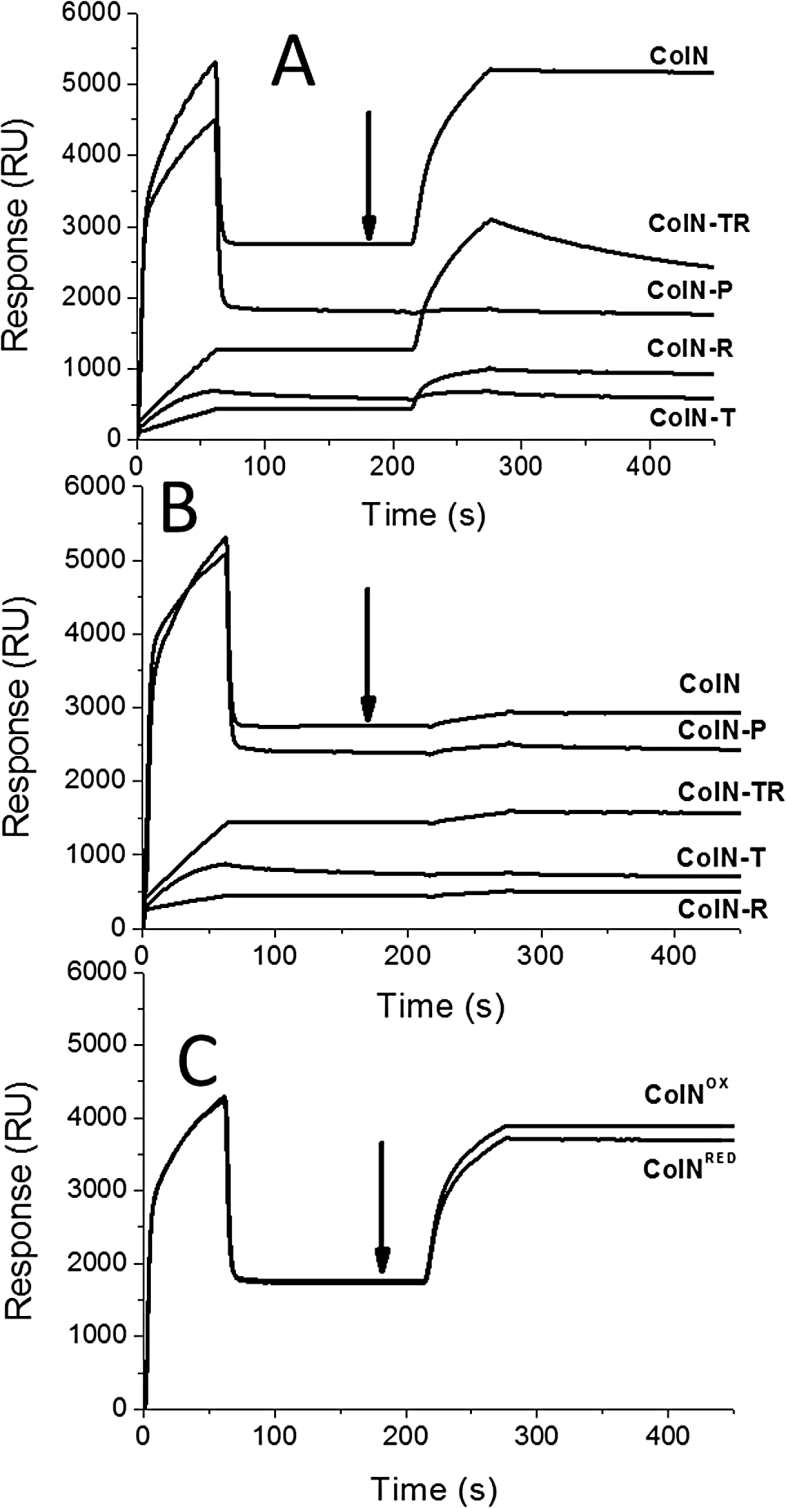
SPR reveals ColN‐R is responsible for LPS binding. Histidine‐tagged ColN domain combinations (500 nM) were injected for 60 s at a flow rate of 5 μl min^−1^ over a Ni^2+^ charged NTA chip at 0 s followed as indicated by black arrows by either (A) 100 μM Rc LPS, (B) 100 μM Rd LPS for 60 s or (C) ColN(V94C/Q179C) in either an oxidized (ColN^OX^) or a reduced (ColN^RED^) state was used. Large drops in signal after ColN construct injections are due to buffer effects. The subsequent differences the quantity of bound material reflected in the different RU values after the binding of different ColN constructs largely correlate with the different masses of the constructs, e.g. ColN 42, ColN‐P 21.5, ColN‐TR 20.5, ColN‐R 11.5 and ColN‐T 9 kDa. The relatively high value for ColN‐T may reflect the denser packing possibly with a natively disordered domain.

SPR was used to identify which regions of ColN were responsible for LPS binding. Various C‐terminally histidine‐tagged ColN domain mutants were injected at equal molar concentrations over a Ni^2+^ charged NTA surface followed by either Rc LPS (Fig. 3A) or Rd LPS (Fig. 3B). The data demonstrate that all R‐domain containing constructs (i.e. ColN, ColN‐TR and ColN‐R) bind to Rc LPS while ColN‐T and ColN‐P do not (Fig. 3A). LPS dissociates more slowly from ColN or ColN‐R than from ColN‐TR which nevertheless binds significant amounts of LPS. Thus ColN‐R serves as the minimal Rc LPS binding unit and it binds with a similar kinetic profile in both the isolated construct and the full‐length ColN protein. In agreement with the genetic screen (Sharma *et al*., 2009) none of the constructs tested bound to Rd LPS (Fig. 3B). All the colicin constructs possess a high net positive charge (pI = ∼ 9.0) and show weak, non‐specific, electrostatic binding to any negatively charged LPS which is revealed by small changes in response units (RUs) with Rd LPS (Fig. 3B) and for ColN‐T and ColN‐P with Rc LPS.

**Figure 4:**
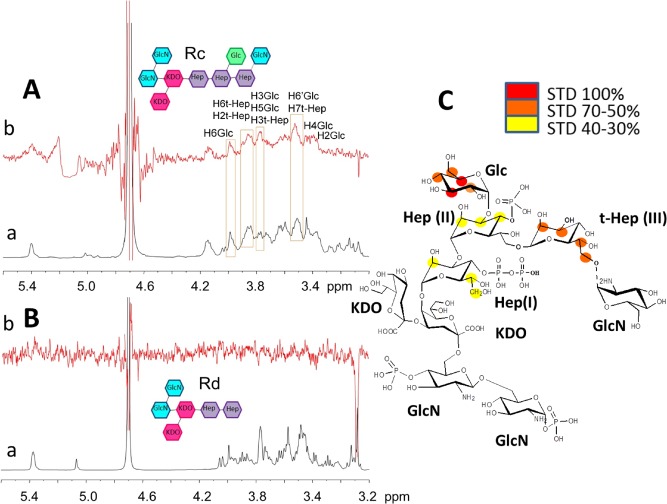
STD NMR data for ColN‐R binding to Rc LPS^Δ^^LIPID^ and Rd LPS^ΔLIPID^. A. Reference ^1^H‐NMR spectrum (a) and 1D STD NMR spectrum (b) of mixed colicin: Rc LPS^ΔLIPID^ 1:100. B. Reference 1H NMR spectrum (a) and 1D STD NMR spectrum (b) of mixed colicin: Rd LPS^ΔLIPID^ 1:100. C. STD‐derived epitope mapping of the ColN‐R:RcLPS^ΔLIPID^ interaction with colour coding from the highest (red) to lowest (yellow) observed STD effect.

In order to probe any conformational changes of ColN upon binding to Rc LPS, Val94 and Gln179 were mutated to cysteine residues in full‐length ColN (Fig. 1C). Oxidation of ColN V94C/Q179C was performed using a mixture of CuSO_4_ (0.2 mM) and 1,10‐phenanthroline (2.0 mM) while irreversible reduction was performed using TCEP (5.0 mM) and iodoacetamide (5.0 mM). An electrophoretic mobility shift on SDS‐PAGE electrophoresis was used to confirm the oxidation/reduction of the disulphide bond (data not shown). SPR was used to assess the *in vitro* effect of locking the R‐domain in ColN. Both oxidized (ColN^OX^) and reduced (ColN^RED^) ColN samples bound Rc LPS similarly when injected at equal molar concentrations, demonstrating that ColN‐R does not require significant unfolding to bind LPS (Fig. 3C).

### STD‐NMR analysis of ColN‐R binding to LPS^Δ^^LIPID^

We used STD‐NMR spectroscopy to investigate the basis of the LPS recognition by ColN‐R. Measuring the transfer of NMR saturation from the protein to the ligand enables the precise mapping of which parts of the ligand are involved in binding (Mayer and Meyer, 1999). As a first step, we acquired STD‐NMR spectra using both Rc LPS^ΔLIPID^ and Rd LPS^ΔLIPID^ as ligands (Fig. 4A and B). Spectra obtained using Rc LPS^ΔLIPID^ confirmed its binding to ColN‐R and indeed a lot of STD enhancements were observed between 4.2 and 3.3 ppm (labelled in Fig. 4A). Overlapping signals impaired the analysis but a qualitative interpretation of the data indicates that the protons in position 3 and 5 of the glucose residue gave rise to strong STD effects indicating that they were involved in the binding process (labelled H3Glc and H5Glc in Fig. 4A). Thus the glucose residue has a key interaction with ColN‐R (Fig. 4A and C). Furthermore STD enhancements of signals belonging to the heptose moieties were also observed and, significantly, the terminal heptose, present in Rc LPS^ΔLIPID^ but not Rd LPS^ΔLIPID^, contributes to the interaction. Identical experiments were performed on Rd LPS^ΔLIPID^ (Fig. 4B) and no STD signals were observed, indicating that there was no binding. These results corroborate the hypothesis that oligosaccharide moieties characteristic of Rc LPS (Glc and HepIII) are fundamental for the recognition and interaction process. The LPS samples used were natural mixtures which tend to show variations in structure which can be defined only by dedicated separation of the components (Klein *et al*., 2013). Thus Fig. 4 represents the most abundant species observed here with a non‐stoichiometric GlcN attached to the terminal heptose in Rc. Also a faint signal from a third Kdo (Frirdich and Whitfield, 2005) (not shown in the figure) was observed.

### ^31^P MAS‐NMR analysis of ColN‐R binding to LPS in membranes

**Figure 5:**
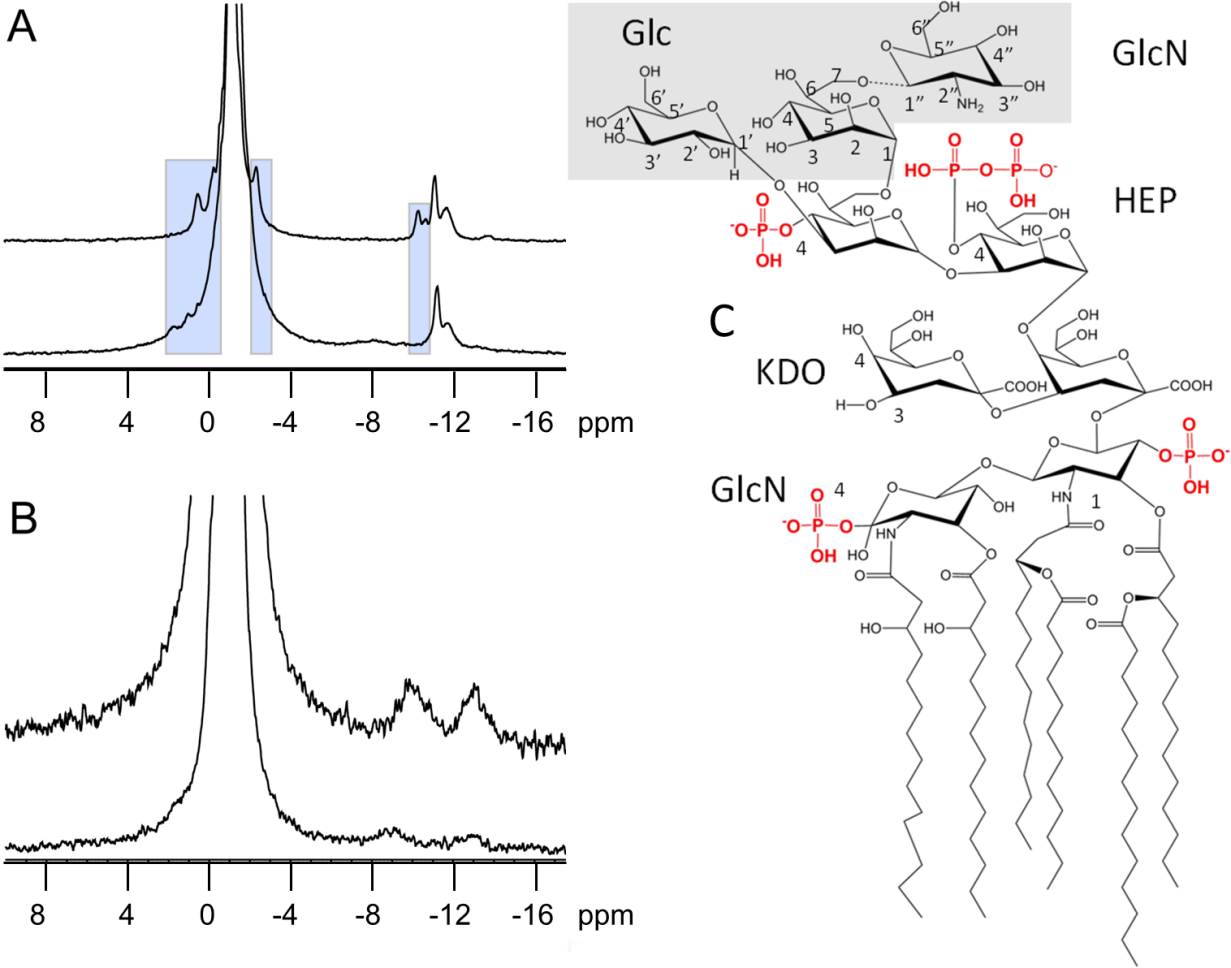
High‐resolution ^31^P MAS‐NMR. A and B. (A) Rc LPS in DOPC membranes in the absence (top) and in the presence (bottom) of ColN‐R; and, from Rd LPS in DOPC membranes (B) in the absence (top) and in the presence (bottom) of ColN‐R. Spectra were acquired at 20°C with 5 kHz sample spinning. The spectral changes due to ColN‐R binding are shaded (see also Table 1). C. The structure of lipid A and core region of LPS is shown for reference with the Rc LPS‐specific sugar residues shaded. Note the dotted line bond connecting the non‐stoichiometric glucosamine to Hep(III). Most assignments described in the text refer to peaks resolved by spectral deconvolutions which are not evident here and are listed in Table 1. The clear peaks in the Rd LPS spectrum (B) are the pyrophosphates on Hep (I) with the phosphate proximal to the pyranose ring at −13 ppm and the proximal one at −9.9 ppm. The extra complexity in this region of the Rc LPS spectrum indicates additional unassigned pyrophosphates probably on the additional t‐Hep, glucose and glucosamine moieties which bind to ColN‐R (see Figs 4C and 5C).

We used high‐resolution, solid‐state ^31^P MAS‐NMR to investigate sites of natural phosphorylation and pyrophosphorylation in Rc and Rd LPS and to follow changes in ^31^P dynamics following addition of ColN‐R. The data were collected in a membrane environment by incorporating Rc LPS (Fig. 5A) and Rd LPS (Fig. 5B) in DOPC membranes (dominant resonance at −1.09 ppm). Phosphate resonances at −0.2 and −0.5 ppm were attributed to Rd LPS, as these were common to both Rc and Rd LPS spectra. These correspond to the single phosphate groups shown in Fig. 5C. The Rc LPS spectra reveal additional phosphorylation sites between −3 and 1 ppm (0.5, −0.2 and −2.3 ppm). Since these are unique to Rc LPS and are absent from Rd LPS, it suggests that pyranose sites on Glc‐(Hep‐II)‐Hep‐GlcN, are available for phosphorylation (Fig. 5C).

**Table 1:** Spectroscopic characteristics from ^31^P MAS‐NMR at 20°C; isotropic chemical shifts, CS_iso_ and resonance intensities normalized to the total spectral intensity

Assignment	Rc‐LPS in DOPC		Rc‐LPS + ColN‐R		Rd‐LPS in DOPC		Rd‐LPS + ColN‐R	
	CS_iso_	Integral	CS_iso_	Integral	CS_iso_	Integral	CS_iso_	Integral
	(ppm)	%	(ppm)	%	(ppm)	%	(ppm)	%
			1.7	1				
			1.01	1				
2 – Rc	0.54	4	0.55	1				
3	−0.06	1						
3 – Rd, Rc	−0.24	2					−0.2	1
4 – Rd	−0.52	2	−0.57	2			−0.52	3
4	−0.66	2						
4	−0.8	1						
DOPC	−1.09	63	−1.19	34	−1.13	94	−1.11	71
Fits	−1.53	6	−1.29	43			−1.71	23
Fits	−1.78	5	−1.6	1				
6 – Rc	−2.31	4	−1.98	2				
PP – Rd^a^			−8.02	10^a^	−9.92	3	−9.09	1
PP – Rc	−10.23	2						
PP – Rc	−10.61	1						
PP – Rc	−11.05	2	−11.17	2				
PP – Rc	−11.62	4	−11.67	1				
PP – Rd	−13.71	2	−13.1	1	−13.01	3	−12.96	1

Resonances were defined by spectral deconvolution (Ciesielski *et al*., 2009; Sanghera *et al*., 2011) and assigned to Rd‐LPS if present at least in the Rd‐LPS spectrum and to Rc‐LPS if present in the Rc‐LPS spectrum only. Positional assignment follows phosphate assignment of pyranose ring phosphates in exopolysaccharide (Dertli *et al*., 2013) and corresponds to the numbering system in Fig. 5. Resonances in Rc LPS most affected by ColN‐R binding are shaded grey.

a Resonance is too broad to quantify accurately.

Pyrophosphorylation resonances are observed between −8 and −15 ppm (Bonev *et al*., 2004; Parisot *et al*., 2008; Ciesielski *et al*., 2013) (Table 1). The resonances between −10 and −12 ppm are unique to Rc LPS while the one at −13.7 ppm has a corresponding resonance in Rd LPS at −13.0 ppm. In the spectrum from Rd we see two resonances from the pyrophosphate at −9.9 and at −13.0 ppm and the two phosphorus resonances have distinct chemical shifts due to the effect of the pyranose on one of them. It is probable that the resonance at −9.9 ppm arises from the distal phosphorus nucleus as in undecaprenyl pyrophosphate (Bonev *et al*., 2004), while the resonance at −13.0 ppm is from the phosphorus adjacent to the pyranose C1 as shown for PP‐1‐Hep(I) (Fig. 5C). This is consistent with slightly longer longitudinal relaxation time T1 of 1.18 s for the −9.9 ppm resonance *versus* 0.99 s for the −13.0 ppm resonance. Overall, we observe significantly more extensive phosphorylation and pyrophosphorylation in the Rc specific Glc‐(Hep)‐Hep‐GlcN region than previously reported (Muller‐Loennies *et al*., 1999) (cf. Fig. 4C). As explained above natural LPS is heterogeneous and this may be reflected in its phosphate content. Furthermore the procedures used to deacylate LPS prior to chemical analysis may remove phosphate groups (Muller‐Loennies *et al*., 1999). A full description of the assignments of these additional signals observed by solid‐state ^31^P MAS‐NMR will be presented elsewhere.

Addition of ColN‐R to Rc LPS‐containing DOPC membranes led to a complete loss of resonance intensity at −10.2 and −10.6 ppm (Fig. 5A, bottom) reflecting a dramatic increase in longitudinal relaxation times, T_1_, for these pyrophosphates due to specific ColN‐R binding to this particular group. Such striking changes in T_1_ (Sanghera *et al*., 2011) are a strong indication that these particular pyrophosphates underpin specific recognition of LPS by ColN‐R. This binding also reduces the intensity of the pyrophosphate resonance at −11.7 ppm relative to that at −11.2 ppm, and suggests proximity of the former to the binding site. Furthermore, it confirms the specificity of the interaction since the latter resonance (−11.2 ppm), is unlikely to be involved directly in ColN‐R binding.

Resonances arising from phosphorylation sites also reflect strong molecular coupling between ColN‐R and Rc LPS as all three phosphate resonances, previously resolved at 0.54, −0.2 and −2.3 ppm, are significantly shifted, broadened or have lost much intensity (Fig. 5A) (Table 1). The observed changes in isotropic chemical shifts are a strong indication of proximity of these phosphorylation sites to the ColN‐R binding epitope. This supports the observation that the Δ*waaP* mutation, which prevents phosphorylation of the initial heptose, abolishes ColN activity (Sharma *et al*., 2009).

Addition of ColN‐R to Rd LPS in DOPC membranes (Fig. 5B, bottom) led to marked reduction in isotropic linewidth of the DOPC resonance and an increase in the effective chemical shift anisotropy, seen as higher intensity in the rotational satellites at ± 5 kHz (data not shown). This is likely to be the result of incorporation of ColN‐R into the membrane, rather than binding to a specific epitope. Reduction in overall DOPC phosphate linewidth in the presence of ColN‐R allows Rd LPS phosphates at −0.20 and −0.52 ppm to be resolved, which we use to assign these to the inner core Rd region of LPS. Residual intensities from librationally broadened resonances can be deconvoluted from the composite spectral intensity observed in the absence of ColN‐R at comparable chemical shifts. This suggests that there are few specific ColN‐R/Rd LPS interactions and any changes are due to membrane‐bound ColN‐R. Importantly, the data confirm that the binding epitope in the membrane agrees with that determined by STD‐NMR.

### ColN‐R requires a minimum oligosaccharide decoration of LPS for binding

**Figure 6:**
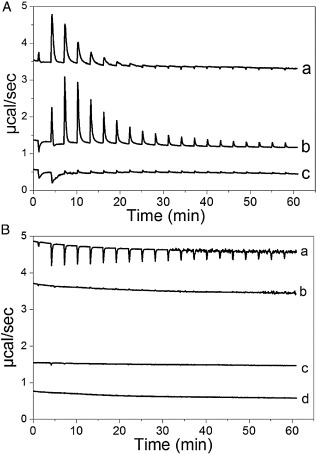
Binding of ColN‐R to LPS and LPS^Δ^^LIPID^ measured by ITC. A. Combined isothermal titration data for the binding of ColN‐R to LPS at 20°C. (a) ColN‐R (1.42 mM) titrated into Rc LPS (0.5 mM), (b) ColN‐R (1.23 mM) titrated into Ra LPS (0.5 mM), (c) ColN‐R (1.23 mM) titrated into Rd LPS (0.5 mM). B. Combined isothermal titration data for the binding of ColN‐R to LPS^ΔLIPID^ at 20°C. (a) ColN‐R titrated into Rc LPS^ΔLIPID^, (b) ColN‐R titrated into buffer control, (c) ColN‐R titrated into Rd LPS^ΔLIPID^, (d) ColN‐R titrated into buffer (control). For all titrations ColN‐R was used at 2.33 mM, LPS^ΔLIPID^ at 0.25 mM. For all LPS samples a concentration of ∼ 1 mg ml^−1^ corresponds to 0.5 mM. Binding isotherms were analysed using the manufacturer's software.

To further test the role of LPS structure on ColN‐R binding we probed, by ITC, its interaction with three different types of LPS; Ra, Rc and Rd (Fig. 6A). To keep ColN‐R in solution the analysis was carried out in buffer containing 150 mM NaCl (Evans *et al*., 1996b). All reactions were performed at 20°C, a temperature at which all LPS samples will be in the gel phase (Brandenburg *et al*., 2005). When ColN‐R was titrated into Rc LPS we observed an endothermic titration which saturated by ∼ the 10th injection (Fig. 6A). A similar titration was observed for the titration of ColN‐R into Ra LPS except that the heat exchanges were moderately higher and the titration was not complete until ∼ the 15th injection (Fig. 6A). The interaction with both Rc and Ra LPS was entropically driven under these conditions and yielded similar *Kd* values, 1.88 and 2.42 μM respectively (Table 2). Attempts were made to study the binding to full‐length ColN to Rc LPS, however, at the 300 mM salt concentration required to keep ColN in solution (Evans *et al*., 1996b) no interaction between ColN and Rc LPS could be detected (data not shown). Similarly, when the ColN‐R:RcLPS interaction was probed in the same high‐salt buffer no interaction was seen, demonstrating the electrostatic component of the interaction (data not shown). In agreement with both the solution and ssNMR, ColN‐R showed no evidence of interaction with Rd LPS (Table 2). In summary ColN‐R binding requires the minimum decoration as seen in Rc LPS but is not hindered if the LPS is extended further as in the case of Ra LPS.

**Table 2:** Thermodynamic analysis of the binding of LPS to ColN‐R

Interaction	*Kd*, μM	N	Δ*H*, kcal·mol^−1^	*T*Δ*S*, kcal·mol^−1^	Δ*G*, kcal·mol^−1^
ColN‐R : Rc LPS	1.88 ± 0.70	N/A	14.65 ± 1.94	22.19 ± 1.84	−7.54 ± 0.10
ColN‐R : Ra LPS	2.42 ± 0.63	N/A	20.15 ± 0.61	27.51 ± 0.56	−7.36 ± 0.05
ColN‐R : Rd LPS	NB	NB	NB	NB	NB

ITC parameters for the binding of Rc, Rd and Ra LPS to ColN‐R at 20°C in 20 mM K^+^ phosphate, pH 7.5, 150 mM NaCl. The errors shown are those from triplicate experiments. NB: no binding (heats of interaction are no greater than heats of dilution). N/A: stoichiometry of binding not applicable to this study. For original data see supplementary information.

### ITC analysis of ColN‐R binding to LPS^ΔLIPID^

In an attempt to resolve the stoichiometry of the ColN–LPS interaction we decided to investigate the binding of ColN‐R to delipidated LPS^ΔLIPID^ by ITC. However this analysis was complicated by the fact we consistently observed protein precipitation in the ITC cell towards the end of the titrations. This only allowed us to qualitatively assess the interaction by ITC (Fig. 6B). However it is clear that ColN‐R does display a much weaker affinity for Rc LPS^ΔLIPID^ than for the lipidated form. Importantly, there was no interaction with Rd LPS^ΔLIPID^, consistent with both the STD‐NMR and ^31^P MAS‐NMR data.

### Potassium efflux measurements clarify the roles of waaG and waaQ

**Figure 7:**
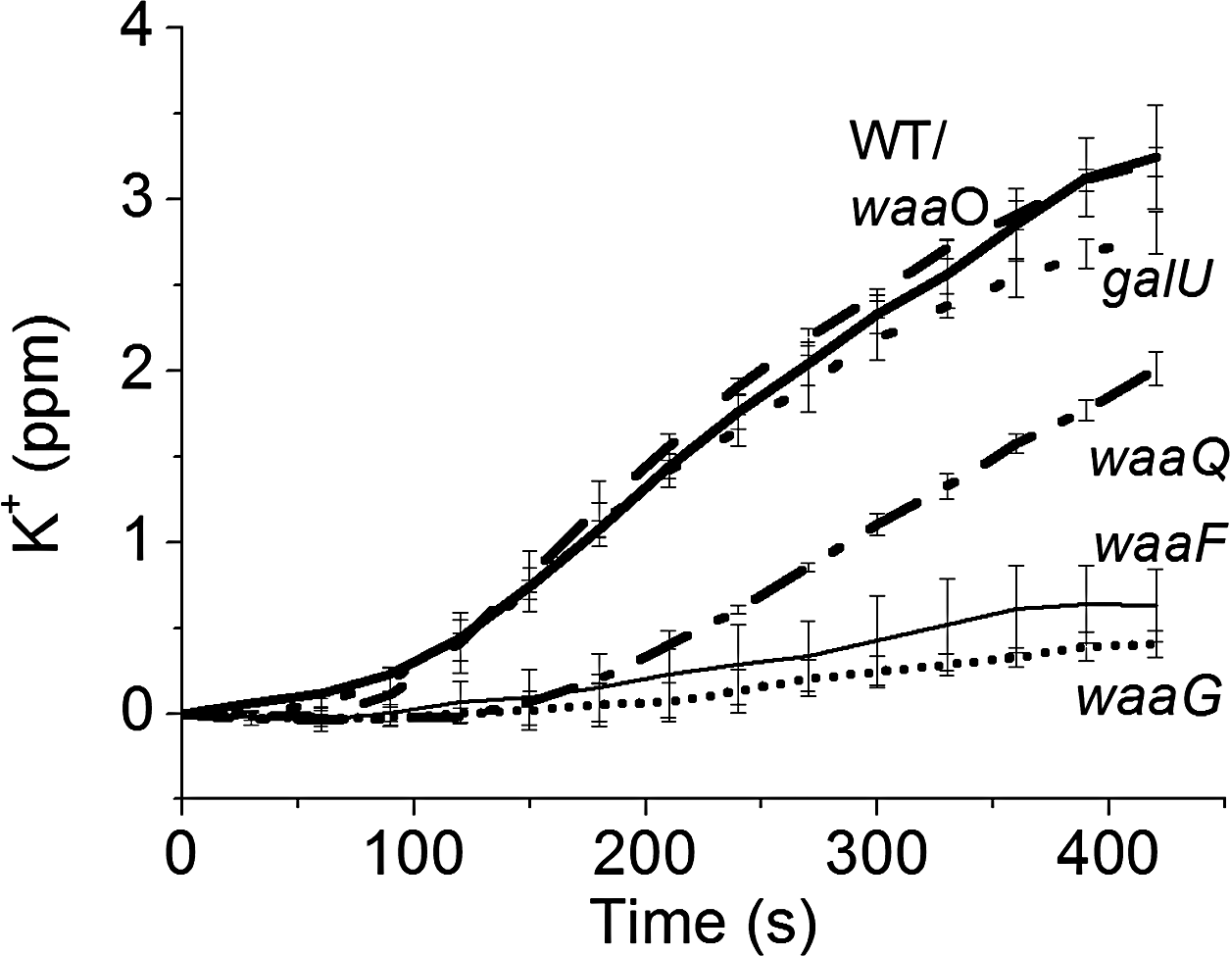
K^+^ efflux measurements using *E**. coli* cells with knockout mutations in the LPS biosynthesis pathway. Cells from the Keio collection were prepared as per *Experimental procedures*. ColN was added at 100 molecules per cell to either wild‐type Keio (dashed), waaO (thick solid), galU (large dots), waaQ (dot dash), waaF (thin solid line), waaG (small dots).

Our *in vitro* data thus confirmed the suggestion of Sharma *et al*. (2009) from growth inhibition assays. To define the *in vivo* effects more precisely we studied the kinetics of ColN activity which can be accurately followed in real time by using an ion‐selective electrode to measure the release of intracellular potassium from affected cells. We used this extremely sensitive assay (Johnson *et al*., 2013) to measure the *in vivo* activity of ColN on various Keio (Baba *et al*., 2006) LPS synthetic pathway mutants. First, the K^+^ efflux measurements confirmed that Δ*waaF* cells, which fail to add the either HepII or HepIII, which we now have shown by STD‐NMR to be involved in ColN‐R binding, were resistant to ColN (Fig. 7). Additionally, K^+^ efflux kinetics revealed subtle effects not observed in the tests of cell growth inhibition employed in the genome‐wide screen (Sharma *et al*., 2009) which found that Δ*galU* and *ΔwaaG* strains both displayed a partial sensitivity to ColN. In K^+^ efflux measurements the *ΔgalU* strains showed almost wild‐type susceptibility to ColN whereas *ΔwaaG* were completely resistant. This is explained by the fact that although GalU and WaaG together add the first glucose to the LPS inner core, GalU, involved in the production of UDP‐glucose, has a leaky phenotype (Qimron *et al*., 2006). On the other hand WaaG uses UDP‐glucose to decorate HepII in the inner core and its absence alone is therefore sufficient to prevent glucose addition and ColN activity. Growth curves of WaaG mutants treated with ColN are unusual as they appear dead then recover after 3 h and then grow well (Sharma *et al*., 2009). To understand why the K^+^ efflux assay shows them to be resistant will need further analysis. The GalU mutant does reduce the K^+^ efflux compared to the WT but the growth curves show a greater reduction in toxicity. Thus intermediate effects seem to influence these assays in different ways yet to be understood. Furthermore, whereas WaaQ, which is involved in the addition of the terminal heptose (t‐Hep/HepIII) to the LPS inner core, was not identified as being important for ColN toxicity by Sharma *et al*. (2009) we found that *ΔwaaQ* cells displayed an intermediate sensitivity to ColN. This agrees precisely with the STD‐NMR data which showed binding to HepIII.

## Discussion

**Figure 8:**
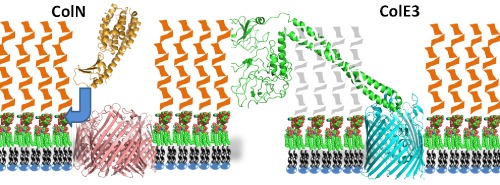
Schematic diagram demonstrating the contrasting receptor binding modes of ColN and ColE3. Diagrams of *E**. coli* outer membranes with LPS in the outer leaflet composed of core LPS (co‐ordinates from FhuA structure 1FCP) and O‐antigen sugars shown schematically. Phospholipids (black chains and blue ovals) in the inner leaflet. OmpF (pink) 2OMF (Cowan *et al*., 1992), and colicin N 1A87 (Vetter *et al*., 1998) are shown with proposed site of interaction with LPS in the outer membrane highlighted by a blue arrow. For ColE3 the model is drawn from PDB files 1JCH (Colicin E3; Soelaiman *et al*., 2001) and 1UJW (Colicin E3 R‐domain and BtuB; Kurisu *et al*., 2003). Since there is no data on E3‐LPS interactions with the O‐antigens this region is greyed out and no assumptions are implied for this part of the model.

All colicins possess a three domain structure with an N‐terminal, usually disordered, translocation or T‐domain, a central receptor binding or R‐domain and a C‐terminal toxic domain which in ColN is a pore‐forming (P‐domain) (El‐Kouhen *et al*., 1993). Group A (Tol‐dependent) colicins A and E1–9 all use the vitamin B12 receptor BtuB (Housden *et al*., 2005; Cascales *et al*., 2007) and high‐resolution co‐crystal structures of E2 and E3 with BtuB show this to be via the tip of the long, coiled‐coil R‐domain (Kurisu *et al*., 2003; Sharma *et al*., 2007) (Fig. 8). This type of interaction is also shown in Group B (Ton‐dependent) colicins by the published structure of ColIa and its receptor Cir (Buchanan *et al*., 2007). Other outer membrane receptors include Tsx (colicins K, 5 and 10), OmpA (U), FepA (B&D), FhuA (M) (Pilsl *et al*., 1999; Cascales *et al*., 2007) and OmpW (S4) (Arnold *et al*., 2009). Most group A colicins need OmpF as well but ColN was unusual in that no high‐affinity primary protein receptor was discovered by genetic analysis and it was assumed to only require OmpF (Bourdineaud *et al*., 1990b; El‐Kouhen *et al*., 1993). ColN was shown to bind directly to OmpF by ITC but a chymotryptic fragment missing the N‐terminal 67 residues of the T‐domain or the isolated R‐domain alone showed much lower affinity (Evans *et al*., 1996a). After the OBS in ColE9 was identified at the tip of the disordered T‐domain (Housden *et al*., 2010), the site of the strong OmpF interaction in ColN was also pinpointed as a linear OBS epitope at the extreme N‐terminus (Johnson *et al*., 2013). However, whereas a point mutation in OBS (F14G) was enough to abolish *in vitro* OmpF binding it hardly affected ColN activity (Johnson *et al*., 2013). Thus OmpF binding by the T‐domain OBS of ColN is not a critical receptor binding step.

Resolution of this conundrum required data from a genome‐wide screen (Baba *et al*., 2006; Sharma *et al*., 2009) which revealed that ColN is unique among the colicins tested in its need for a defined minimum intact LPS biosynthesis pathway. Furthermore, since LPS elongation is a sequential process, genetic deletions showed that a minimum length of LPS, equal to the Rc form, is needed for toxicity. The results presented here clarify the role of LPS binding in the translocation of ColN across the outer membrane by defining the physical interaction of ColN with LPS.

Fortunately the ColN‐R ‘receptor binding domain’ does not need to be renamed since in this article the study of truncated ColN molecules by SPR revealed that this domain is responsible for LPS binding. So, although the abundant LPS receptor is very different from the more usual single high‐affinity protein receptor, the position of the R‐domain within the colicin molecule remains the same. The LPS molecules used in this study are from rough mutants which lack the long water‐soluble O‐antigen (Lindberg and Hellerqvist, 1971). As a result they behave similarly to phospholipids in solution and therefore will form liposome like structures (Le Brun *et al*., 2013). This concentration of the LPS into dense membranes makes mass transport a limiting variable in the SPR analysis which is likely to artificially reduce the observed off rates of the interactions. Thus it was not possible to measure binding affinities by kinetic analysis. However SPR, being a surface‐based technique allowed us to compare the binding ability of each of the domains under identical buffer conditions (i.e. 150 mM salt). This is important, as to keep ColN in solution at useable concentrations usually requires 300 mM NaCl which we know from ITC prevents binding of ColN‐R to Rc LPS. By using SPR, ColN could be bound at low density to the NTA surface, circumventing its aggregation in solution. This allowed us to show that it binds specifically to Rc but not Rd LPS in 150 mM NaCl. Since the minimal ColN‐R domain appears to contain the complete LPS binding site and only required 150 mM NaCl for solubility, we used this version in subsequent ITC measurements.

This ITC analysis confirmed that ColN‐R binds both Rc and Ra but not Rd LPS. However it cannot be relied upon to give an accurate ColN‐R:LPS binding stoichiometry since, being in a bilayer‐like phase, only a fraction of the LPS is available on the outer leaflet for binding (Heerklotz, 2004). Furthermore, the large size of ColN‐R may occlude several LPS molecules without binding to them. For this reason in Table 2 we have not quoted the apparent stoichiometries. The charge neutralization model used for binding peptides such as polymyxin (Brandenburg *et al*., 2005) is also unlikely to be applicable since the fixed 3D structure of ColN‐R cannot expose all the positively charged residues to the anionic LPS surface unless there was a substantial structural rearrangement upon LPS binding, which we have shown, by SPR and cysteine mutagenesis, does not take place. This hurdle could be overcome using the soluble oligosaccharide portion of LPS (LPS^ΔLIPID^). Use of Rc and Rd LPS^ΔLIPID^ did confirm that the core region is responsible for the specificity however the binding of these soluble molecules was too weak to allow for full analysis. The higher affinity of ColN‐R for entire LPS may be due to a direct interaction with the acyl chains or a general interaction with the dense LPS in the membrane phase as observed in the ^31^P MAS‐NMR experiments using Rd LPS‐DOPC membranes (Fig. 4). In either case it is unsurprising considering that ColN‐R has evolved to recognize LPS in the membrane.

Rc LPS used here differs from Rd LPS by an additional glucose and terminal heptose attached to a non‐stoichiometric glucosamine and the presence of at least the first two are critical for binding to LPS. This selectivity was clearly shown by the STD NMR data which showed the strongest effects for the glucose and HepIII moieties which are unique to Rc LPS^ΔLIPID^. Sensitive K^+^ efflux measurements of *in vivo* activity have for the first time highlighted the *in vivo* requirement of the terminal heptose (mediated by WaaQ) to confer full susceptibility. WaaQ was not identified as important in the original screen; however, our data are consistent with the STD‐NMR findings that the glucose along with terminal Hep‐III are the key binding epitopes present in Rc LPS. SS‐^31^P‐MAS‐NMR was used to probe the binding of ColN‐R to Rc and Rd LPS in a defined membrane phase and again confirmed the ability of ColN‐R to discriminate between Rc and Rd LPS. Further work is needed to conclusively assign the positions of the interacting phosphates on the LPS since the inhibition by salt and the Δ*waaP* mutation (Sharma *et al*., 2009) indicate they are essential for binding.

The combined genetic and biophysical results confirm a clear and specific interaction of ColN‐R with the core region of LPS and force a re‐evaluation of the role of OmpF/ColN‐R interactions. While the OBS site in the T‐domain may account for the observed ITC and some OmpF channel blocking data there is clear evidence for ColN‐R (i.e. lacking OBS) binding to OmpF in membranes (Stora *et al*., 1999) and in whole cells (Bourdineaud *et al*., 1990b; El‐Kouhen *et al*., 1993; Evans *et al*., 1996b). In the former it is shown that, while R‐domain can block OmpF channels in a tethered lipid bilayer, the affinity is 100‐fold less for detergent solubilized OmpF, which also explains the weak binding by ITC (Evans *et al*., 1996b). In the whole cells data it is important to note that in pull‐down experiments ColN‐R does not bind to OmpF free *E. coli* B‐ or K‐12 strains although they do possess the required LPS. The inability of LPS alone to bind ColN in these assays might be explained by the presence of 300 mM (Evans *et al*., 1996b) or 160 mM (El‐Kouhen *et al*., 1993) NaCl but in any case the data imply a direct interaction between ColN‐R and OmpF in membranes. Another interpretation is that the gap in the LPS layer that OmpF creates may expose the required LPS binding site at the protein lipid interface (Fig. 8) and enable a strong interaction which can even be seen in higher salt concentrations. Interestingly the interaction with LPS is not hindered by the addition of further glucose moieties to the chain as ColN‐R is equally capable of interacting with both Ra LPS and Rc LPS. Thus ColN targets the base of LPS which is close to the OmpF surface. Since LPS is always co‐purified tightly bound to OmpF (Holzenburg *et al*., 1989; Baboolal *et al*., 2008) it may also be possible that *in vitro*, as well as *in vivo*, binding may involve an OmpF‐LPS complex. Combined with ColN's ability to displace tightly bound LPS from the outside of the OmpF trimer (Baboolal *et al*., 2008). this binding could position the colicin for further translocation across the outer membrane in a manner fundamentally different from other colicins (Fig. 8) (Clifton *et al*., 2012).

## Experimental procedures

### Sample preparation

LPS (Sigma Aldrich) samples were prepared in 20 mM K^+^ phosphate, pH 7.5, 150 mM NaCl, typically at a concentration of 0.5 mM (assuming a Mr of 2000 Da) (Blume and Garidel, 1999). Once resuspended, samples were sonicated on ice, centrifuged for 10 min at 13 000 *g* and the supernatant temperature cycled between 4°C and 70°C before storage at 4°C overnight before use the following day.

### Protein purification

DNA sequences encoding the constructs used in this work were synthesized by GeneArt (Regensburg, Germany). In addition to encoding the colicin constructs in the forward frame the cognate colicin N immunity protein was encoded in the reverse frame. As a contrast to (Evans *et al*., 1996b; Fridd *et al*., 2002), which used N‐terminal histidine tags, all the GeneArt DNA sequences were synthesized with a C‐terminal –SSHHHHHH tag with Nde1 (5′) and BamH1 (3′) restriction sites for subcloning into pET3a (Novagen). Originally the R‐domain was defined as starting at residue 67 which corresponded to a chymotryptic fragment (El‐Kouhen *et al*., 1993) but when the X‐ray structure was solved (Vetter *et al*., 1998)we redefined the R‐domain as the structured region starting at residue 90. The constructs sizes used here are shown in Fig. 1 and ColN‐R, ColN‐RP and ColN‐P had an additional start methionine at the N‐terminus. All colicin constructs were purified as described previously (Fridd *et al*., 2002), followed by dialysis into 50 mM sodium phosphate, pH 7.6, 300 mM NaCl. The disulphide mutant ColN V94C/Q179C was selected as close enough to form a disulphide bond by visual inspection of the X‐ray structure of colicin N (1A87; Vetter *et al*., 1998) since the method of Hazes and Dijkstra (1988) did not select sites in the flexible N‐terminal region that locked large sections. Otherwise the method was similar to that previously described for colicin A (Duché *et al*., 1994). Disulphide bond formation was confirmed by altered migration on SDS‐PAGE (see *Results*).

### Isothermal titration calorimetry (ITC)

ITC measurements were performed using a MicroCal ITC_200_ thermostated at 20°C, with all samples prepared in 20 mM phosphate buffer, pH 7.5, 150 mM NaCl. LPS was present in the sample cell at a concentration of 0.5 mM (assuming a molecular weight of 2000 Da) with the ColN‐R concentration in the syringe varying from 1.23 to 1.42 mM. For ITC experiments involving LPS^ΔLIPID^ ColN‐R was used at 2.33 mM and LPS^ΔLIPID^ at 0.25 mM. Binding isotherms were analysed using the manufacturer's software and fitted to a one‐site binding model. As observed previously isotherms in the gel phase were complex with early data points being less endothermic (Brandenburg *et al*., 2005; Garidel *et al*., 2008). These points were not included in the analysis (see supplementary data Figs S1–S3 and Tables S1–S3). Such effects might be avoided by conducting titrations at 40°C or 45°C when a pure liquid crystalline state is achievable (Garidel *et al*., 2008)

### K^+^ efflux assay using Keio strains

A single colony of *E. coli* cells was used to inoculate 5 ml of 1× LB and grown overnight, shaking at 37°C. After 16 h growth 1 ml of cells was used to inoculate 100 ml of 1× LB supplemented with 10 mM KCL. Cells were grown by shaking at 37°C, until an OD_600_ of 0.5–0.6. Cells were harvested by centrifugation at 3000 *g* at 25°C for 15 min. The supernatant was carefully discarded the cell pellet washed with 4 × 1 ml aliquots of 100 mM sodium phosphate, pH 7.0 before resuspension in 1 ml of 100 mM sodium phosphate, pH 7.0, 5% glycerol. Cells were stored on ice and used within 3 h of preparation after allowing for 30 min equilibration on ice. Following equilibration a defined aliquot containing 5 × 10^9^ cells was taken and added to a jacketed K^+^ efflux vessel maintained stirring at 37°C containing 6 ml of pre‐warmed assay buffer. Data recording was started upon addition of cells using an ion‐selective K^+^ electrode, double junction lithium acetate reference electrode and a temperature probe. Upon addition to vessel the cells re‐accumulate K^+^ over approximately 5 min until a stable baseline is reached. Unless otherwise stated colicin was added to the vessel 60 s after the cells had reached a stable baseline. Data were normalized to zero at the point of colicin addition and measurements continued for 7 min, with data points being taken every 5 s. For all K^+^ efflux measurements the basal rate of K^+^ loss from 5 × 10^9^ cells was recorded to serve as a negative control. The results shown are representative of a minimum of three independent experiments.

### LPS de‐acylation

In order to obtain delipidated polysaccharides, aliquots of both Rc and Rd LPSs were dissolved in anhydrous hydrazine (1 ml per 20 mg sample), stirred at 37°C for 90 min, cooled, poured into ice‐cold acetone (10 ml ml^−1^ Hydrazine), and allowed to precipitate. The precipitate was then centrifuged (3000 *g*, 30 min), washed twice with ice‐cold acetone, dried, dissolved in water and lyophilized. Both products were de‐N‐acylated with 4 M KOH, 120°C, 18 h as described (Molinaro *et al*., 2002) Salts were removed by gel permeation chromatography with Sephadex G‐10 (Pharmacia) column (50 × 1.5 cm) to yield the resulting oligosaccharide.

### STD‐NMR

All NMR experiments were performed on a Bruker 600‐MHz DRX equipped with a cryo probe at 283 K. All the samples were 50 mM sodium phosphate, pH 7.4, 150 mM NaCL. STD NMR experiments were recorded with 32k data points and zero filled up to 64k data points prior to processing; in order to increase the S/N ratio the FIDs were multiplied with an exponential function (l b = 1–2 Hz). Reference STD NMR spectra on both protein and ligands in the free state were performed at different irradiation frequencies, in order to avoid artefacts due to false‐positive STD signals. For the STD spectra acquired on the bound ligands the pseudo 2D pulse program stddiff.3 was used and the unwanted broad resonance signals of the protein were avoided by using a spin lock pulse of 50 ms. For protein saturation a 40 Gauss pulse with a length of 50 ms and an attenuation of 60 db was used. The ‘on resonance’ frequency was set at –0.5 ppm since no STD ligand signals were observed in the corresponding reference spectrum, while 40 ppm was set as the off resonance pulse frequency. For the mixtures, STD NMR experiments were performed using ColN‐R:LPS^ΔLIPID^ molar ratio which was varied from 1:50 to 1:100 and saturation time of 2 s. The STD effects were calculated by (I0 − Isat)/I0, where (I0 – Isat) is the intensity of the signal in the STD NMR spectrum and I0 is the peak intensity of an unsaturated reference spectrum (off‐resonance). The STD signal with the highest intensity was set to 100% and the others were normalized to this peak. Data acquisition and processing were performed with TOPSPIN software.

### Solid‐state NMR

All solid‐state NMR measurements were performed on a Varian VNMRS‐400 MHz spectrometer using a 4 mm T3 MAS NMR probe (Varian/Agilent) with vortex tube‐regulated temperature (Ciesielski *et al*., 2009). HEPES 20 mM pH 7.5 replaced the phosphate buffer used elsewhere. Direct excitation at 104 kHz field was used to observe ^31^PMAS‐NMR spectra under 45 kHz SPINAL 64 heteronuclear proton decoupling (Fung *et al*., 2000). The interpulse delay was set to 5 s to allow recovery of magnetization and 8192 transients were averaged to obtain each spectrum. Longitudinal relaxation times, T_1_, were determined at 2°C and 20°C using inversion recovery, as previously described in LPS studies (Ciesielski *et al*., 2013) and, apart from the Rc LPS polyphosphate (see results), did not exceed 2 s. Thus the repeat delay was set to 5 s, which exceeds fivefold T_1_ for most common biological phosphates. Relaxation times were obtained assuming a single dominant relaxation mechanism. Spectra were processed with ADC. Labs (Advanced Chemistry Development, Toronto, Canada). Homogeneous line broadening was assumed and resonance lines were approximated with mixed Gauss‐Lorentz lineshapes.

### SPR measurements

All analyses of interactions between ColN domain mutants and Rc/Rd LPS were performed on a BIAcore X100 system equipped with a NTA sensor chip (Biacore GE Healthcare) at a flow rate of 5 μl min^−1^. For immobilization of proteins, the chip was treated with 500 μM NiCl_2_ for 1 min before a 60 s injection of purified protein (500 nM). Then 100 μM Rc or Rd LPS was injected for 60 s and the dissociation followed. In order to continuously monitor the non‐specific background binding of samples to the Ni^2+^ surface, LPS was injected over a control flow cell which lacked protein. Regeneration of the chip surface required a 180 s injection of 10 mM HEPES pH 8.3, 150 mM NaCL, 350 mM EDTA followed by a 60 s injection of 100 mM NaOH. All measurements were performed at 25°C in buffer containing 10 mM HEPES pH 7.5, 150 mM NaCl, 50 μM EDTA. Data were analysed with BIAcore X100 evaluation software ver.1.0 (Biacore GE Healthcare). In all cases results presented are representative of a minimum of triplicate experiments.

## Supplementary Material

Supporting Information
