# Supplementary material for: The antibacterial toxin colicin N binds to the inner core of lipopolysaccharide and close to its translocator protein
Source: Mol Microbiol. 2014 Mar 28;92(3):440–52. doi: 10.1111/mmi.12568 (PMC4114557; doi:10.1111/mmi.12568)
Supplement: Supplementary file 1 — Supporting Information [file mmi-92-440-s9.pdf]

Supplementary data for "The antibacterial toxin Colicin N binds to the inner core of lipopolysaccharide and close to its protein translocon."

Christopher L. Johnson[1], Helen Ridley[1], Roberta Marchetti[2], Alba Silipo[2], David C. Griffin[3], Lucy Crawford [1], Boyan Bonev[3], Antonio Molinaro[2], Jeremy H. Lakey[1]*

[1]Centre for Bacterial Cell Biology, Institute for Cell and Molecular Biosciences, Faculty of Medical Sciences, Newcastle University, Framlington Place, Newcastle- upon-Tyne, NE2 4HH, UK. [2] Department of Organic Chemistry and Biochemistry, University of Naples Federico II, Via Cinthia 4, 80126 Napoli, Italy. [3] School of Life Sciences, University of Nottingham, Queens Medical Centre, Nottingham, NG7 2UH, UK.

* e-mail: jeremy.lakey@ncl.ac.uk

Results of isothermal titration calorimetry measurements of R-domain binding to intact (e.g. not delipidated) Ra, Rc or Rd LPS .
Supporting data for table 2 of main paper.

ColN-R titrated into Rc LPS

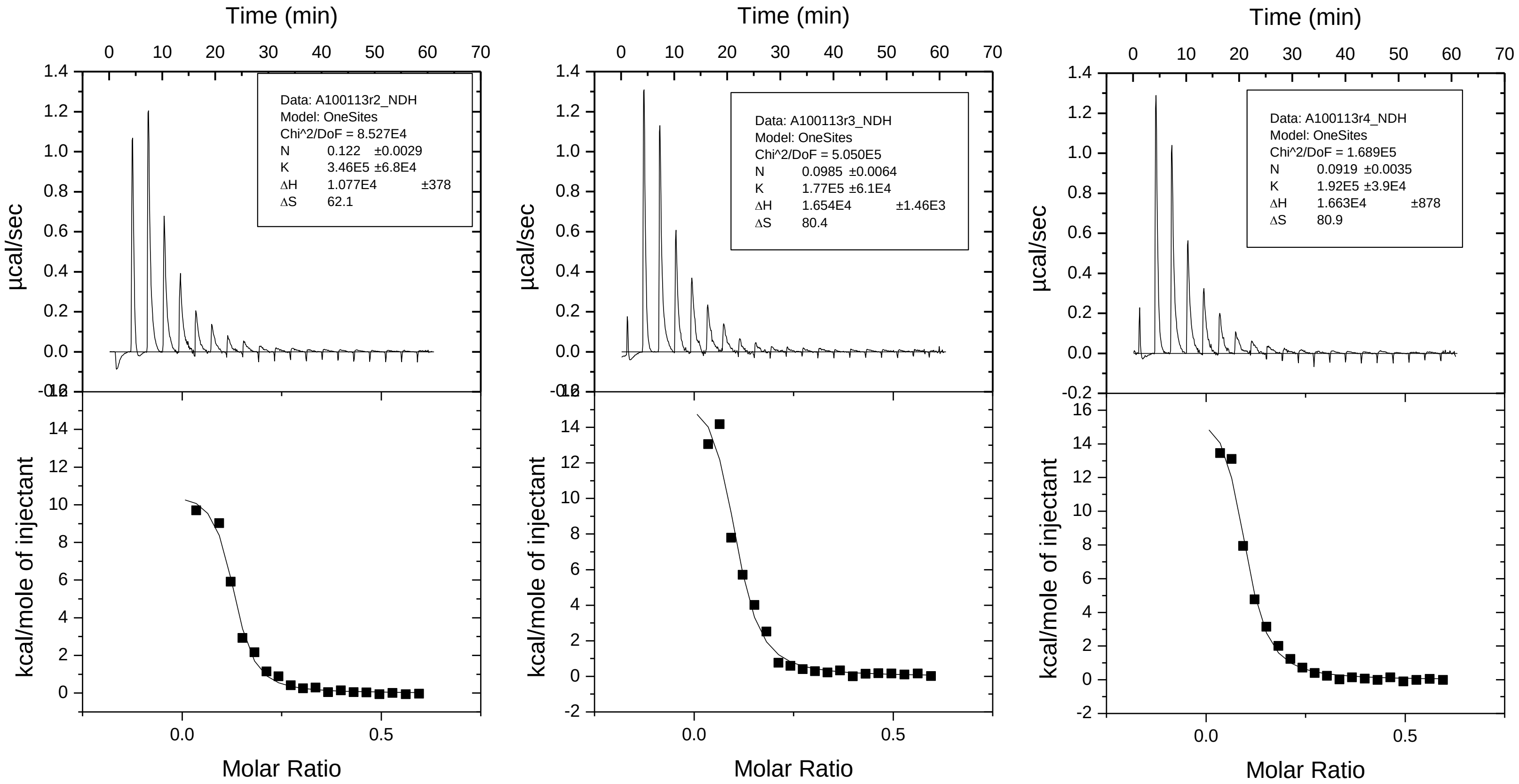

Figure S1: Triplicate titrations of ColN-R (1.42 mM) into Rc LPS (0.5 mM) Buffer = 20 mM potassium phosphate pH 7.5, 150 mM NaCl. Temp = 20°C

	Kd, µM	N	*ΔH*, kcal.mol $^{-1}$	*TΔS,* kcal.mol $^{-1}$	*ΔG*, kcal.mol $^{-1}$
R-domain – Rc LPS	1.88 ± 0.70	N/A	14.65 ± 1.94	22.19 ± 1.84	-7.54 ± 0.10

Table S1. Thermodynamic analysis of the binding of ColN-R to Rc LPS. The errors shown are those from triplicate experiments. Stoichiometry (N) is not applicable due to the uncertainty of available Rc LPS in micellar form.

ColN-R titrated into Ra LPS

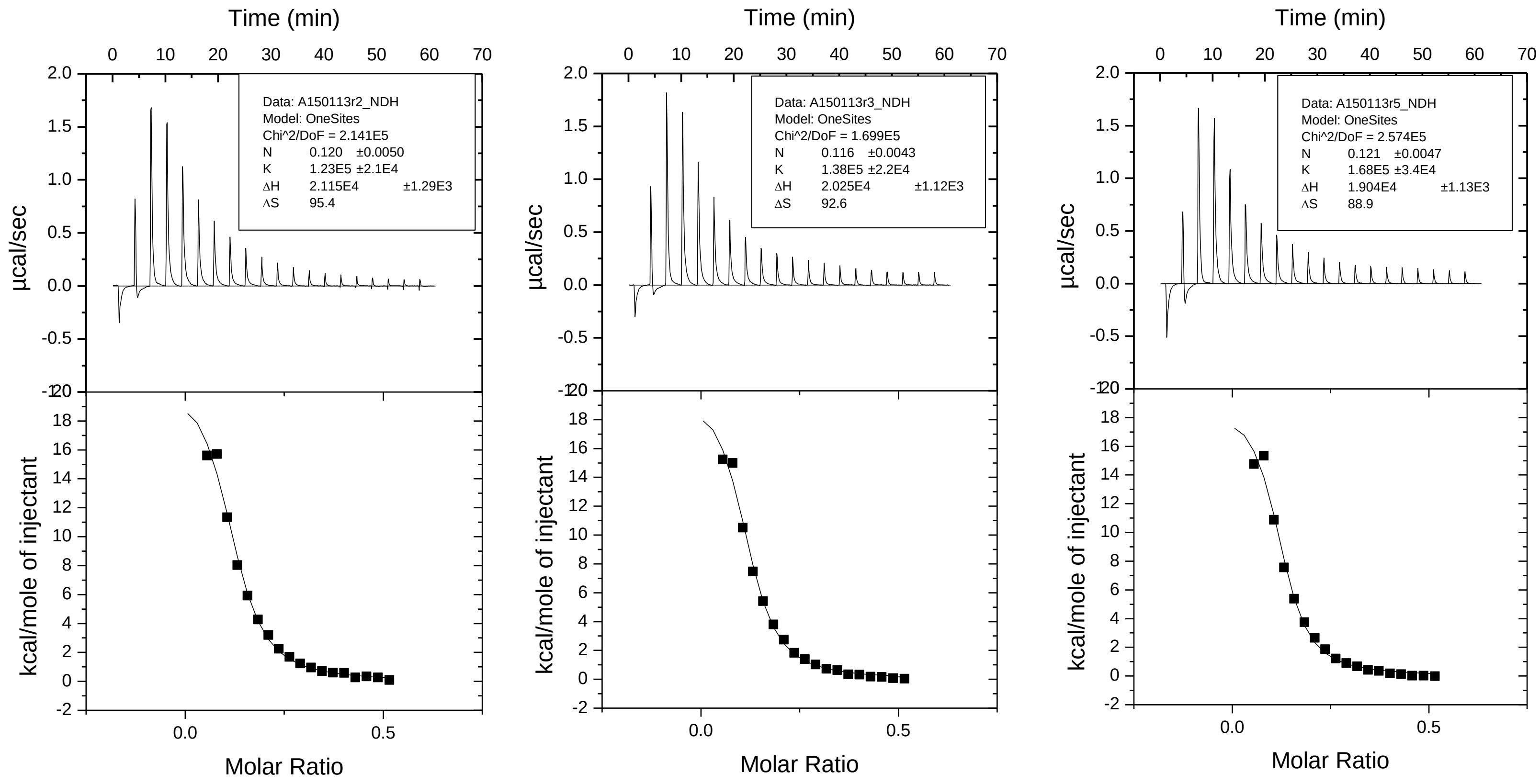

Figure S2: Triplicate titrations of ColN-R (1.23 mM) into Ra LPS (0.5 mM) Buffer = 20 mM potassium phosphate pH 7.5, 150 mM NaCl. Temp = 20°C

	Kd, µM	N	*ΔH*, kcal.mol $^{-1}$	*TΔS,* kcal.mol $^{-1}$	*ΔG*, kcal.mol $^{-1}$
R-domain – Ra LPS	2.42 ± 0.63	N/A	20.15 ± 0.61	27.51 ± 0.56	-7.36 ± 0.05

Table S2. Thermodynamic analysis of the binding of ColN-R to Ra LPS. The errors shown are those from triplicate experiments. Stoichiometry (N) is not applicable due to the uncertainty of available Rc LPS in micellar form.

ColN-R titrated into Rd LPS

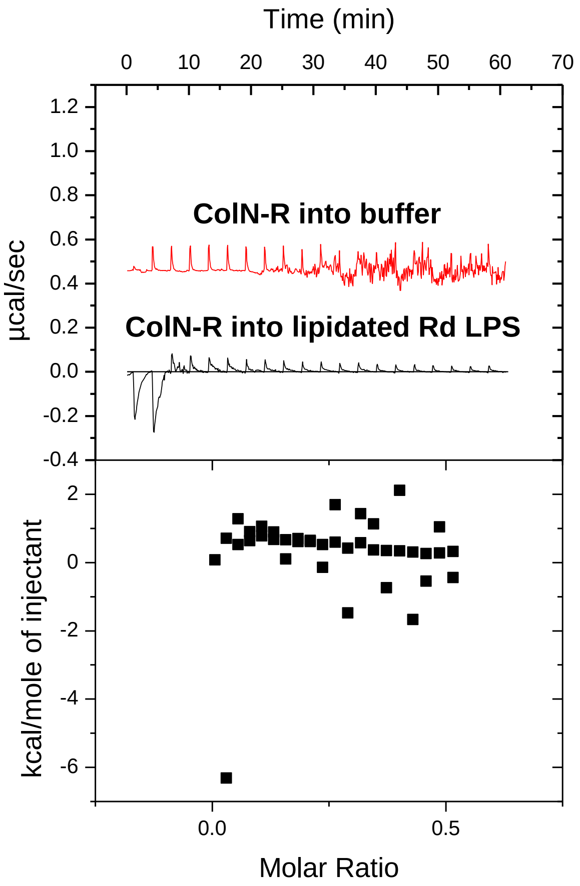

Figure S3: Triplicate titrations of ColN-R (1.23 mM) into Rd LPS (0.5 mM) Buffer = 20 mM potassium phosphate pH 7.5, 150 mM NaCl. Temp = 20°C

	Kd, µM	N	*ΔH*, kcal.mol $^{-1}$	*TΔS*, kcal.mol $^{-1}$	*ΔG*, kcal.mol $^{-1}$	*Source*
R-domain – Rd LPS	NB	NB	NB	NB	NB	180213

Table S3. Thermodynamic analysis of the binding of ColN-R to Rd LPS. The errors shown are those from triplicate experiments. NB = no binding (heats of interaction are no greater than heats of dilution)
